# Beyond glycemia: Comparing tirzepatide to GLP-1 analogues

**DOI:** 10.1007/s11154-023-09825-1

**Published:** 2023-08-01

**Authors:** John Andraos, Harleen Muhar, Shawn R. Smith

**Affiliations:** 1 College of Pharmacy, Western University of Health Sciences, 91766 Pomona, CA USA; 2 Touro College of Pharmacy, 94592 Vallejo, CA USA

**Keywords:** Tirzepatide, Incretin, Twincretin, Type 2 Diabetes, Weight Loss, GLP-1/GIP receptor agonist

## Abstract

Glucagon-like peptide-1 receptor analogs (GLP-1 RAs) have been an innovative and instrumental drug class in the management of both type 2 diabetes and obesity. Tirzepatide is a novel agent that acts as an agonist for both GLP-1 receptors and gastric inhibitory polypeptide (GIP) receptors, another incretin that lowers glucose and appetite. Although previous studies showed a lack of therapeutic benefit for GIP agonists, current studies show that the glucose lowering and weight loss effects of tirzepatide are at least as effective as GLP-1 RAs with a similar adverse effect profile. Some studies, though not conclusive, predict that tirzepatide may in fact be more potent than GLP-1 RAs at reducing weight. A thorough review of the studies that led to tirzepatide’s approval allows for comparisons between tirzepatide and GLP-1 RAs; it also allows for predictions of tirzepatide’s eventual place in therapy - an agent used preferentially over GLP-1 RAs in patients with or without diabetes desiring to lose weight.

## Introduction/background: role of incretins in diabetes and weight

**Fig. 1:**
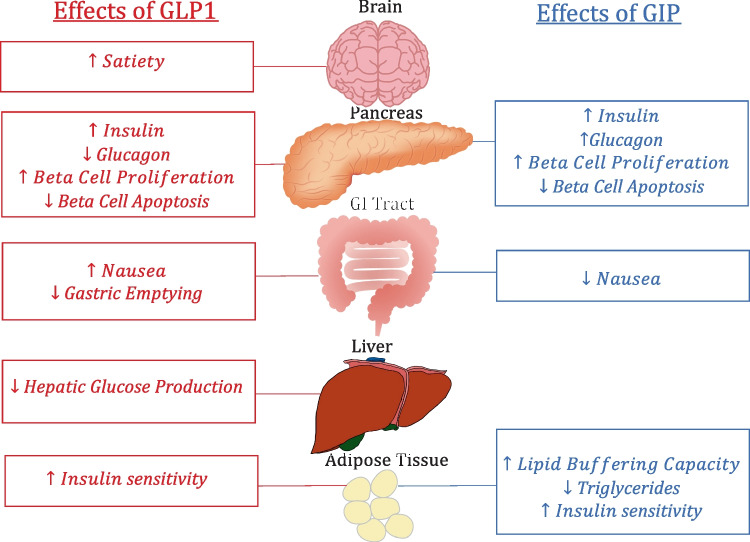
Highlights the relevant functions of both GLP1 and GIP on the body

Incretins are endogenous proteins responsible for multiple bodily functions including the moderation of blood glucose and the feelings of satiety [1]. After being produced by enteroglucagon-producing L cells in the lower gut in response to food intake, incretins travel to various receptors including the pancreas and cause glucose-dependent insulin release causing lowering of elevated blood glucose [1]. Incretins are also responsible for other functions both inside and outside the pancreas. The main functions of incretins are shown in Fig. 1 and are summarized in detail below.

The 2 major incretins found in the body are gastric-inhibitory-polypeptide (GIP) and glucagon-like-peptide-1 (GLP-1) [2]. Glucagon-like-peptide-2 (GLP-2) is a similar compound that is typically not considered an incretin due to its lack of glycemic effects. Though similarities exist in their structure and function, each incretin has some differing effects. In order to lower glucose, GIP travels to beta cells in the pancreas and activates GIP receptors (GIPr) via class-II G-protein coupled receptors causing an increase in intracellular cAMP and calcium leading to a release of insulin [3]. Though the exact mechanism is unclear, GIP levels can become raised without inducing hypoglycemia in normoglycemic patients [4]. GIP’s other functions include fat metabolism and promoting beta cell proliferation [5]. The hormone also decreases the parietal cell’s secretion of gastrin and stomach acid, proliferates the bone’s osteoblasts and inhibits osteoclasts, and may be involved in neuro-signaling as evidenced by widespread expression of GIPr in the brain. Though their function is unclear, GIP receptors are also present in other organs such as the heart, kidneys, and skin [6].

Similarly, GLP-1 binds to GLP-1-receptors (GLP-1r) and causes the same glycemic effect as GIP on the pancreas. Agonists attach to G-protein coupled receptors causing an increase in intracellular cAMP and calcium leading to a release of insulin [7]. Other effects of GLP-1 include inhibiting gastric emptying, decreasing food intake, and decreasing glucagon secretion resulting in a decrease in endogenous glucose production [7–9]. GLP-1 has also been shown to protect beta cells from apoptosis and stimulate beta-cell proliferation [10, 11]. The mechanisms of these effects are largely unknown.

Much of the appetite suppression studies have occurred with GLP-1 which causes appetite suppression in a multifactorial way. First, GLP-1 causes an increase in satiety and a decrease in hunger leading to decreased food intake [11]. Additionally, incretins seem affect the vagus nerve in the central nervous system (CNS) to decrease GI motility decelerating gastric emptying [12]. This leads to a delayed feeling of fullness and decreased acid production. It is proposed that GIP works similarly but may also affect lipid metabolism enhancing the weight loss effects [13]. GIP affects the ability of the body to expand its white adipose tissue storage in a healthy way allowing for increased lipid breakdown and decreased lipid spillover and ectopic fat accumulation in various organs [13]. Decreasing ectopic lipid accumulation also may promote insulin sensitivity [13].

GLP-2’s effect predominantly resonates in the gut and the protein does not seem to directly affect glucose levels [14]. Although each of these peptides differ from each other, similarities also exist. Apart from being produced in similar locations and having some overlapping functions, all three of these proteins are traditionally not orally bioactive [4]. Recently, however, semaglutide, a GLP-1 receptor analog (GLP-1 RA), has been formulated in a way to allow for oral absorption [15]. Additionally, they are all broken down similarly by dipeptidyl peptidase-4 (DPP-4) though GIP breakdown occurs at a slower rate than GLP-1 breakdown [16].

## Introduction/background: history of incretin mimetics medications

GIP was the first incretin isolated and characterized in the 1970’s though most modern therapies focus on GLP-1 [17]. This was because the function of GIP in the pathophysiology of diabetes was historically questionable. Most scientists previously believed that GLP-1 levels were deficient in patients with diabetes [18]. Though recent studies show this may not be true, the belief led to the development of agents to overcome GLP-1 deficiencies [19].

On the other hand, GIP deficiency did not seem to be pathogenic of diabetes leading to the peptide being mostly ignored after initially being discovered and isolated [20]. Further evidence has increased the understanding of GIP showing that GIP likely still has a role in diabetes despite normal or sometimes elevated concentrations. This research theorized that hyperglycemia can lead to a downregulation of GIPr causing decreased GIP response despite therapeutic or even supratherapeutic levels of GIP [21]. Patients with diabetes seem to have a lower response to GIP regardless of the concentration of the hormone [21]. This discouraged researchers from pursuing GIP as a therapeutic target. GIP supplementation was also shown to be ineffective in the 1990s when intravenous (IV) GIP was introduced to hyperglycemic patients with no effect on their blood glucose though physiologic rather than pharmacologic concentrations were supplied [20]. However, when the experiments were done with GLP-1, blood glucose was reduced [22].

Prior to formulating incretin mimetics, two administration issues had to be resolved - their lack of oral bioavailability and their short half-lives [23]. The aforementioned studies were completed through IV administration of proteins which is not feasible in the treatment of a chronic disease. Another barrier is that following production in the body, DPP-4 rapidly breaks down incretins. In fact, DPP-4 inhibitors, aimed at elongating effect of endogenous incretins, are available for the treatment of type 2 diabetes though the glycemic effect of these medications are comparatively modest [24]. Thus, in order to have GLP/GIP analogs with lasting effects, alterations of the peptides needed to be made in order to prevent their rapid metabolism by DPP-4 and oral administration could not be used.

Subcutaneous injections resolved the oral bioavailability issues of incretins while the short half-life barrier was resolved when researchers identified other animals that also produce similar GLP-1 molecules [25]. The salivary glands of gila monsters produce a GLP-1 peptide that does not have a DPP-4-recognized site leading to the first therapeutic incretin, exenatide - a twice daily subcutaneous injection [25]. The phase 3 trials showed not only success in the lowering of glucose, but also the reduction in weight. Other manufacturers began synthesizing GLP-1 analogs with altered DPP-4 binding sites that bypassed degradation and remained in the body for weeks at a time [26]. A longer-acting version of exenatide (weekly), liraglutide (daily), dulaglutide (weekly), and both injectable (weekly) and oral (daily) semaglutide were approved in the coming years. [27–31]. Other GLP-1 RA have also been approved but either no longer exist on the market or are not as frequently used. This novel class of medications currently yields one of the largest reductions in A1C [32]. Since the medication causes glucose-dependent insulin release and did not cause hypoglycemia but suppressed appetite, liraglutide and semaglutide were both approved for weight loss in patients without diabetes [33, 34].

Another significant benefit of the medication class is its ability to reduce cardiovascular risk in patients with type 2 diabetes. Several of the medications showed that patients with a history of cardiac disease or risk factors for cardiovascular disease had lower risks of heart attacks, strokes, and myocardial infarctions when taking these agents compared to patients taking standard of care regardless of A1C lowering [35–37].

The American Diabetes Association now recommends GLP-1 RAs for patients with established ASCVD regardless of their A1C and regardless of if the patient is taking metformin, the historically first line medication for T2DM [38]. The guidelines also recommend using the medication in patients with a compelling need to minimize weight gain, patients prone to hypoglycemia, and patients who need a substantial A1C reduction [38]. The guidelines support the medication class’s positive effects of slowing the progression of chronic kidney disease though the effects are likely not as strong as SGLT2i, another class of diabetes medications [39, 40]. Alternatively, the guidelines recommend choosing alternative glucose lowering therapies if cost is a major issue for a patient as these brand-name medications are highly priced [38].

As previously mentioned, two GLP-1 RAs are available for the management of weight even in patients who do not have diabetes [33, 34]. GLP-1 RAs are one of the most potent weight-loss agents while also not being stimulatory like most other weight loss medications. However, these agents can be expensive for patients whose insurances do not cover the medications.

Following the successes of GLP-1 RAs, manufacturers have worked to produce similar outcomes with newer agents that affect the incretin pathways including not only GLP1 receptors but also GIP and glucagon receptors. Though several molecules, such as mazdutide, a GLP/glucagon dual agonist, are being studied in various phases, the first agent targeting incretins other than just GLP-1 was approved in 2022 – tirzepatide [41].

## Introduction/background: a novel dual incretin analog

Despite historical research stating GIP did not assist in lowering blood glucose even with supratherapeutic levels, the success of GLP-1 RAs has led to the approval of a novel GLP-1/GIP RA, tirzepatide [42]. The medication is a single molecule that targets both incretin receptors, GLP-1r and GIPr. When activated with GLP-1r, GIP seems to potentiate not only the weight loss mechanism of the incretin but also the glucose lowering effects [13]. GIP activation may additionally potentiate the insulin secretion effects of GLP-1 activation on the pancreas and the weight loss effects of GLP-1 activation on CNS [13]. GIP activation may also provide complimentary mechanisms by potentiating the expansion of white adipose tissue and lipid handling [13]. Finally, GIPr agonism may decrease the nauseating effects of GLP-1 agonism through area postrema inhibitory neurons potentially allowing for higher therapeutic doses of GLP-1 analogs with decreased adverse effects [13].

### Tirzepatide effects: glucose lowering effects of tirzepatide

Tirzepatide, a once-weekly subcutaneous injection, was FDA approved in May 2022 as an adjunct treatment with diet and exercise to improve glycemic control in adults with Type 2 diabetes [43]. Its approval was preceded by the outcomes of five phase III trials, which are summarized in Tables 1 and 2, each of which compare tirzepatide at 5 mg, 10 mg, and 15 mg to other medications comparing similar clinical endpoints. These trials demonstrated its efficacy and safety in Type 2 diabetes with or without other anti-diabetic agents [42, 44–47]. Apart from characteristics noted in the study designs, baseline traits including age, race, and gender were similar among all of the SURPASS trials and did not impact outcomes [42, 44–47]. In depth inclusion and exclusion criteria are also included in Table 1. SURPASS-1 shows that tirzepatide alone, at all doses, was superior to placebo and revealed a dose-dependent reduction of HbA1c by up to 2.07% (p < 0.0001) from baseline to week 40 [47]. SURPASS-3, -4, and -5 are comparisons of HbA1c reduction between tirzepatide and other anti-diabetic agents [42, 44, 45]. When trialed in patients who were already taking either insulin degludec or glargine, tirzepatide achieved about double the percentage of A1C lowering effects (difference of up to 1.14%) [42, 45]. Tirzepatide is currently being studied in an ongoing trial as an alternative to prandial insulin in patients currently on a basal insulin to treat type 2 diabetes [48].

**Table 1:** Inclusion and Exclusion Criteria of SURPASS Trials

Study	Inclusion Criteria	Exclusion Criteria
SURPASS-1	• Have been diagnosed with type 2 diabetes mellitus (T2DM). • Are naïve to diabetes injectable therapies and have not used any oral antihyperglycemic medications (OAMs) during the 3 months preceding screening. • Have HbA1c between ≥7.0% and ≤9.5%. • Be of stable weight (± 5%) for at least 3 months before screening. • Have a BMI ≥23 kilograms per meter squared (kg/m^2^) at screening.	• Have type 1 diabetes mellitus. • Have had chronic or acute pancreatitis any time prior to study entry. • Have proliferative diabetic retinopathy or diabetic maculopathy or nonproliferative diabetic retinopathy requiring acute treatment. • Have disorders associated with slowed emptying of the stomach, or have had any stomach surgeries for the purpose of weight loss. • Have an estimated glomerular filtration rate <30 mL/minute/1.73 m^2^. • Have had a heart attack, stroke, or hospitalization for congestive heart failure in the past 2 months. • Have a personal or family history of medullary thyroid carcinoma or personal history of multiple endocrine neoplasia syndrome type 2. • Have been taking weight loss drugs, including over-the-counter medications during the last 3 months.
SURPASS-2	• Have been diagnosed with type 2 diabetes mellitus (T2DM) • Have HbA1c between ≥7.0% and ≤10.5% • Be on stable treatment with unchanged dose of metformin >1500 mg/day for at least 3 months prior to screening • Be of stable weight (±5%) for at least 3 months before screening • Have a body mass index (BMI) ≥25 kilograms per meter squared (kg/m^2^) at screening	• Have type 1 diabetes mellitus • Have had chronic or acute pancreatitis any time prior to study entry • Have proliferative diabetic retinopathy or diabetic maculopathy or nonproliferative diabetic retinopathy requiring acute treatment • Have disorders associated with slowed emptying of the stomach, or have had any stomach surgeries for the purpose of weight loss • Have acute or chronic hepatitis, signs and symptoms of any other liver disease, or blood alanine transaminase (ALT) enzyme level >3.0 times the upper limit of normal (ULN) for the reference range, as determined by the central laboratory. Participants with nonalcoholic fatty liver disease (NAFLD) are eligible for participation in this trial only if there ALT level is ≤3.0 the ULN for the reference range • Have an estimated glomerular filtration rate <45 milliliters/minute/1.73 m^2^ (or lower than the country specific threshold for using the protocol required dose of metformin per local label) • Have had a heart attack, stroke, or hospitalization for congestive heart failure in the past 2 months • Have a personal or family history of medullary thyroid carcinoma or personal history of multiple endocrine neoplasia syndrome type 2 • Have been taking any other diabetes medicines other than metformin during the last 3 months • Have been taking weight loss drugs, including over-the-counter medications during the last 3 months
SURPASS-3	• Have been diagnosed with type 2 diabetes mellitus (T2DM) • Have HbA1c between ≥7.0% and ≤10.5% • Be on stable treatment with unchanged dose of metformin or metformin plus an SGLT-2 inhibitor for at least 3 months before screening • Be of stable weight (± 5%) for at least 3 months before screening • Have a BMI ≥25 kilograms per meter squared (kg/m2) at screening	• Have type 1 diabetes mellitus • Have had chronic or acute pancreatitis any time prior to study entry • Have proliferative diabetic retinopathy or diabetic maculopathy or nonproliferative diabetic retinopathy requiring acute treatment • Have disorders associated with slowed emptying of the stomach, or have had any stomach surgeries for the purpose of weight loss • Have acute or chronic hepatitis, signs and symptoms of any other liver disease, or blood alanine transaminase (ALT) enzyme level >3.0 times the upper limit of normal (ULN) for the reference range, as determined by the central laboratory. Participants with nonalcoholic fatty liver disease (NAFLD) are eligible for participation in this trial only if their ALT level is ≤3.0 the ULN for the reference range • Have an estimated glomerular filtration rate <45 mL/minute/1.73 m2 (or lower than the country specific threshold for using the protocol required dose of metformin per local label) • Have had a heart attack, stroke, or hospitalization for congestive heart failure in the past 2 months • Have a personal or family history of medullary thyroid carcinoma or personal history of multiple endocrine neoplasia syndrome type 2 • Have been taking any other diabetes medicines other than metformin, or metformin plus an SGLT-2 inhibitor during the last 3 months • Have been taking weight loss drugs, including over-the-counter medications during the last 3 months
SURPASS-4	• Have been diagnosed with type 2 diabetes mellitus (T2DM) • Have HbA1c between ≥7.5% and ≤10.5% • Be on stable treatment with unchanged dose of at least 1 and no more than 3 types of oral antihyperglycemic drugs, which may only include metformin, SGLT-2 inhibitors, and/or sulfonylureas for at least 3 months before screening • Have increased risk for cardiovascular (CV) events • Be of stable weight (± 5%) • Have a BMI ≥25 kilograms per meter squared (kg/m2) at screening	• Have type 1 diabetes mellitus • Have had chronic or acute pancreatitis any time prior to study entry • Have proliferative diabetic retinopathy or diabetic maculopathy or nonproliferative diabetic retinopathy requiring acute treatment • Have disorders associated with slowed emptying of the stomach, or have had any stomach surgeries for the purpose of weight loss • Have acute or chronic hepatitis, signs and symptoms of any other liver disease, or blood alanine transaminase (ALT) enzyme level >3.0 times the upper limit of normal (ULN) for the reference range, as determined by the central laboratory. Participants with nonalcoholic fatty liver disease (NAFLD) are eligible for participation in this trial only if their ALT level is ≤3.0 the ULN for the reference range • Have an estimated glomerular filtration rate <45 mL/minute/1.73 m2 (or lower than the country specific threshold for using the protocol required dose of metformin per local label) • Have had a heart attack, stroke, or hospitalization for congestive heart failure in the past 2 months • Have a personal or family history of medullary thyroid carcinoma or personal history of multiple endocrine neoplasia syndrome type 2 • Have been taking any other diabetes medicines other than metformin, or metformin plus an SGLT-2 inhibitor during the last 3 months • Have been taking weight loss drugs, including over-the-counter medications during the last 3 months
SURPASS-5	• Have been diagnosed with type 2 diabetes mellitus (T2DM) and have been treated with insulin glargine (U100), once daily with or without metformin ≥3 months prior to screening visit. • Have HbA1c between ≥7.0% and ≤10.5%. • Have a stable weight (± 5%) for at least 3 months before screening. • Have a body mass index (BMI) ≥23 kilograms per meter squared (kg/m^2^) at screening.	• Have type 1 diabetes mellitus. • Have had chronic or acute pancreatitis any time prior to study entry. • Have proliferative diabetic retinopathy or diabetic maculopathy or nonproliferative diabetic retinopathy requiring acute treatment. • Have disorders associated with slowed emptying of the stomach, or have had any stomach surgeries for the purpose of weight loss. • Have an estimated glomerular filtration rate <30 mL/minute/1.73 m^2^ [for participants on metformin, estimated glomerular filtration rate <45 mL/min/1.73 m2 (or lower than the country-specific threshold for using the protocol-required dose of metformin per local label)] • Have had a heart attack, stroke, or hospitalization for congestive heart failure in the past 2 months. • Have a personal or family history of medullary thyroid carcinoma or personal history of multiple endocrine neoplasia syndrome type 2. • Have been taking weight loss drugs, including over-the-counter medications during the last 3 months.
SURPASS-6	• Have been diagnosed with type 2 diabetes mellitus (T2DM) • Have HbA1c between ≥7.5% and ≤11% • Have been treated for at least 90 days prior to day of screening with once or twice daily basal insulin with or without stable dose of metformin ≥1500 mg/day and up to maximum approved dose per country specific approved label, sulfonylureas or dipeptidyl peptidase 4 inhibitors • Be of stable weight (± 5%) for at least 90 days • Have a BMI ≥23 kilograms per meter squared (kg/m^2^) and ≤45 kg/m^2^ at screening	• Have type 1 diabetes mellitus • Have had chronic or acute pancreatitis any time prior to study entry • Have proliferative diabetic retinopathy or diabetic macular edema or non-proliferative diabetic retinopathy requiring immediate or urgent treatment • Have disorders associated with slowed emptying of the stomach, have had any stomach surgeries for the purpose of weight loss, or are chronically taking drugs that directly affect gastrointestinal motility • Have had a heart attack, stroke, or hospitalization for congestive heart failure in the past 2 months • Have a personal or family history of medullary thyroid carcinoma or personal history of multiple endocrine neoplasia syndrome type 2 • Have been taking weight loss drugs, including over-the-counter medications during the last 3 months • Have an estimated glomerular filtration rate <30 mL/minute/1.73 m^2^ [for participants on metformin, estimated glomerular filtration rate <45 mL/min/1.73 m2 (or lower than the country-specific threshold for using the protocol-required dose of metformin per local label)]

[44–49]

Tirzepatide was also compared to GLP-1 RA’s. SURPASS-2 studied tirzepatide versus 1 mg semaglutide, a once-weekly injectable GLP-1 RA, in patients taking metformin monotherapy [46]. Over 40 weeks, the estimated difference between the 15 mg tirzepatide group and the semaglutide group was -0.45 percentage points (95% CI, -0.57 to -0.32; P<0.001). Tirzepatide showed further efficacy in A1c lowering when all doses outperformed placebo and higher doses (10 and 15 mg) outperformed dulaglutide 1.5 mg by 1.1%, another once-weekly GLP-1r injectable in a phase II randomized-controlled trial [50]. Since the trials, higher doses of dulaglutide (3 mg, 4.5 mg) and semaglutide (2 mg) have become available. Still, the ADA has included tirzepatide in its list of agents with “very high efficacy” along with semaglutide and high dose dulaglutide [38].

**Table 2:** Comparison of Tirzepatide to Other Antidiabetic Agents

***Trial***	***Sample size (n)***	***Trial duration (weeks)***	***Tirzepatide doses (mg)***	***Comparator***	***Additional Anti-diabetic regimen***	***Mean treatment difference in A1c versus comparator***	***Mean treatment difference in weight versus placebo***
**SURPASS-1**	478	40	5 10 15	Placebo	None	–1∙91% (95% CI –2∙18 to –1∙63), –1∙93% (–2∙21 to –1∙65), –2∙11% (–2∙39 to –1∙83) (all P<0∙0001)	–6∙3 kg (95% CI –7∙8 to –4∙7), –7∙1 kg (–8∙6 to –5∙5), –8∙8 kg (–10∙3 to –7∙2) (all P<0∙0001)
**SURPASS-2**	1,879	40	5* 10 15	Semaglutide 1 mg	Metformin	−0.15 % (95% CI, −0.28 to −0.03; P=0.02), −0.39 % (−0.51 to −0.26; P<0.001), −0.45 % (−0.57 to −0.32; P<0.001)	−1.9 kg (95% CI, −2.8 to −1.0), −3.6 kg ( −4.5 to −2.7), −5.5 kg (−6.4 to −4.6) (all P<0.001)
**SURPASS-3**	1,444	52	5 10 15	Insulin degludec	Metformin +/- SGLT2 inhibitor	-0.59% (95% CI, -0.73 to -0.45), -0.86% (-1.00 to -0.72), -1.04% (-1.17 to -0.90), (All P<0.001)	-9.8 kg (95% CI, -10.8 to -8.8), -13.0 kg (-14.0 to -11.9), -15.2 kg (-16.2 to -14.2), (All P < 0.001)
**SURPASS-4**	2,002	52	5 10 15	Insulin glargine	Metformin +/- sulfonylurea +/- SGLT2 inhibitor	-0.80% (97.5% CI, -0.93 to 0.66) -0.99% (-1.13 to -0.86), -1.14% (-1.28 to -1.00), (All P < 0.001)	-9.0 kg (95% CI, -9.8 to -8.3), -11.4 kg (-12.1 to -10.6), -13.5 kg (-14.3 to -12.8), (All P < 0.001)
**SURPASS-5**	475	40	5 10 15	Placebo	Insulin glargine +/- Metformin	-1.30% (95% CI, -1.52 to -1.07), -1.66% (-1.88 to -1.43), -1.65% (-1.88 to -1.43), (All P < 0.001)	-7.8 kg (95% CI, -9.4 to -6.3), -9.9 kg (-11.5 to -8.3), -12.6 kg (-14.2 to -11.0), (All P < 0.001)
**SURPASS-6**	1,428	Ongoing	5, 10, 15	Insulin lispro	Insulin glargine +/- Metformin	Ongoing	Ongoing

[44–49]

### Tirzepatide effects: weight loss effects of tirzepatide

Aside from assessing glucose-lowering effects in the SURPASS trials, tirzepatide was studied for weight loss and obesity in the SURMOUNT trials. SURMOUNT-1 assessed the efficacy and safety of tirzepatide compared to placebo in people with a BMI of 30 or more or a BMI of 27 with at-least one weight related complication, excluding diabetes. Tirzepatide was dosed at 5 mg, 10 mg, and 15 mg. The mean percentage of change in weight from baseline was 15.9%, 19.5%, and 20.9% (p < 0.001) respectively when compared with placebo. The trial showed that tirzepatide not only causes a dose-dependent lowering of blood glucose, but also a dose-dependent lowering of weight [48]. SURMOUNT-2 reached completion in April 2023 and aimed to assess the efficacy and safety in patients with both obesity and type 2 diabetes. This trial evaluated the use of 10 mg and 15 mg tirzepatide in 938 adult participants. Tirzepatide achieved up to 15.7% weight loss compared to placebo though the data evaluation of the trial is still in progress; the manufacturer will continue to evaluate the results and present them in the coming months. [51]. Tirzepatide’s approval for weight loss is anticipated though the results of SURMOUNT -3 and -4 trials are also being conducted.

Weight loss was also measured as a secondary endpoint in the SURPASS trials (also included in Table 2). In the trials versus insulin, the tirzepatide group achieved approximately 7-10 kg weight loss compared to approximately 2.3 kg weight gain in the insulin group (p < 0.0001) [44]. Though direct comparisons are lacking, tirzepatide seems to be more potent at lowering weight than any other weight loss medication available. In SURPASS-2, tirzepatide 15 mg resulted in a 11.2 kg reduction in body weight versus 5.7 kg with 1 mg semaglutide (p < 0.001) [46]. Similarly, the higher doses of tirzepatide resulted in greater weight loss than dulaglutide [50]. Again, new doses of semaglutide and dulaglutide have since been approved and direct comparisons of these higher doses to tirzepatide are lacking. Additionally, the comparative studies were done only in patients with diabetes limiting the generalizability of any conclusions made.

Table 3 lists FDA-approved weight loss medication regardless of diabetes status along with their average reductions of body weight. Prior to tirzepatide being on the market, semaglutide showed the largest amount of placebo-subtracted weight loss in comparison to other drugs. In addition to glucose lowering and weight loss, it is important to note that tirzepatide is a non-sympathomimetic drug, unlike some other FDA-approved weight loss drugs containing phentermine and bupropion. ADA 2023 emphasizes the importance of losing weight stating >10% weight loss can be disease modifying and may even lead to disease remission [38]. The SURPASS and SURMOUNT trials have led the ADA to consider tirzepatide, along with semaglutide, to be “very highly efficacious for weight loss” [38].

**Table 3:** FDA Approved Weight Loss Medications and Their Efficacies

***Drug***	***Year Approved***	***Dosage***	***Dosage form***	***Placebo-subtracted weight loss (%)***
Semaglutide	2021	2.4 mg once weekly	Injection	12.4
Phentermine-Topiramate	2012	15/92 mg once daily	Oral	8.6
Liraglutide	2014	3 mg daily	Injection	5.4
Bupropion-naltrexone	2014	360/32 mg once daily	Oral	4.8
Phentermine	1959	30 mg once daily	Oral	4.4
Orlistat	1999	120 mg three times a day	Oral	3.8

[52]

### Tirzepatide effects: cardiovascular, renal, hepatic, and other metabolic effects of tirzepatide

Due to concerns that type 2 diabetes medications increased cardiovascular risk, the FDA issued guidance for pharmaceutical companies to perform cardiovascular outcomes trials for new diabetes medications. As mentioned previously, liraglutide, dulaglutide, albiglutide, and semaglutide have been shown to reduce the risk of cardiovascular events in patients with diabetes and either established ASCVD or high-risk characteristics in randomized controlled trials [35, 36, 49]. Diabetes guidelines recommend these GLP-1 RA’s as pharmacologic agents in patients with ASCVD or with high-risk indicators for cardiovascular disease. These agents are integral in clinical practice to improve cardiovascular outcomes in patients with diabetes.

A meta-analysis of 42,920 participants from five randomized trials demonstrated that GLP-1 RA’s are associated with a significant reduction in the risk of the composite cardiovascular disease in patients with diabetes and established ASCVD [53]. While the impact of GLP-1 RA’s on cardiovascular outcomes has been shown to be directly correlated with the reduction in A1c, these agents exhibit pleiotropic effects [54]. These include reduction of systolic and diastolic blood pressure, body weight, vascular inflammation, and improvement of endothelial function [55–58]. Additionally, a phase 2 trial of patients with type 2 diabetes has shown tirzepatide improves cardiovascular biomarkers [59, 60].

The SURPASS-4 trial is the first randomized controlled trial assessing cardiovascular outcomes with Tirzepatide [45]. Tables 1 and 2 summarized the details of the study along with the glucose-lowering effects of tirzepatide as compared to insulin glargine. Additionally, the study looked at cardiovascular outcomes as a pre-specified secondary analysis which included a four-point composite endpoint of cardiovascular death, myocardial infarction, stroke, and hospitalization for unstable angina. Adjudicated MACE-4 events occurred in 109 participants and were not increased on tirzepatide compared with glargine (HR 0·74, 95% CI 0·51–1·08). These results suggest there is no excess cardiovascular risk with tirzepatide and are consistent with the improvements of numerous surrogate markers of cardiovascular health, including weight reduction, glycemic control with less hypoglycemia, blood pressure reduction, and improvements in the lipid profile. While SURPASS-4 was not powered to evaluate differences in MACE-4 incidence between tirzepatide treatment groups and glargine, it was designed to fulfill the MACE-4 non-inferiority analysis requested by regulatory authorities to evaluate the cardiovascular safety of new glucose lowering medications.

Sattar et al. conducted a pre-specified cardiovascular meta-analysis that included all seven randomized controlled trials with a duration of at least 26 weeks from the SURPASS trials [61]. The primary objective of this meta-analysis was the comparison of the time to first occurrence of confirmed four-component major adverse cardiovascular events including cardiovascular death, myocardial infarction, stroke and hospitalized unstable angina between tirzepatide groups and control groups. 4,887 participants treated with tirzepatide and 2,328 control participants were analyzed and 142 participants had at least one MACE-4 event. The HRs comparing tirzepatide versus controls were 0.80 (0.57–1.11) for MACE-4; 0.90 (0.50–1.61) for cardiovascular death; and 0.80 (0.51–1.25) for all-cause death. The cardiovascular safety demonstrated by tirzepatide in this analysis and SURPASS-4 paved the way for a large-scale cardiovascular outcomes trial.

SURPASS-CVOT is a phase 3 study enrolled approximately 13,000 patients at least 40 years of age and have a diagnosis of type 2 diabetes, confirmed atherosclerotic cardiovascular disease, HbA1c ≥7.0% to ≤10.5% and a body mass index (BMI) ≥25 kilograms per meter squared (kg/m^2^). Patients were excluded if they had a major cardiovascular event within the last 60 days, type 1 diabetes mellitus, history of pancreatitis, or a history of severe hypoglycemia. The primary endpoint is a three-point major adverse cardiovascular event endpoint that includes death from cardiovascular causes, non-fatal myocardial infarction (MI), or nonfatal stroke over a study period of 54 months. The study compares tirzepatide with the long-acting GLP-1 RA, dulaglutide for non-inferiority and superiority, and is currently in progress [61]. SURPASS-CVOT will not compare tirzepatide to placebo as commonly done. SURPASS-CVOT is unique, in that it compares the dual incretin agonist with a GLP-1 RA that has been shown to have cardiovascular benefit in people with type 2 diabetes and high risk ASCVD. The results of the SURPASS-CVOT will define the impact of tirzepatide on cardiovascular disease. Currently, the ADA does not include tirzepatide as part of their agents with cardiorenal benefit [38].

In addition to cardiovascular and renal effects, tirzepatide has shown promising hepatic effects. In a 26-week study, tirzepatide was studied to determine its effect on biomarkers of nonalcoholic steatohepatitis (NASH) and fibrosis in patients with T2DM. When compared to dulaglutide and placebo, tirzepatide statistically significantly displayed improvement in ALT and AST from baseline. Other NASH-related biomarkers were decreased, and adiponectin was increased in patients with T2DM [62].

SURPASS-3 MRI, a sub-study of the phase-3 SURPASS-3 trial assessed tirzepatide administered once per week versus insulin degludec on liver fat content (LFC). A total of 296 participants without a history of significant alcohol consumption were randomly assigned to active treatment. The study looked at the change from baseline in LFC as assessed by MRI-proton density fat fraction (MRI-PDFF) at week 52. The absolute reduction in LFC at week 52 was significantly greater for the tirzepatide 10 mg and 15 mg group versus the insulin degludec group. The reduction in LFC was significantly correlated (p≤0.0006) with baseline LFC (ρ=-0.71), reductions in visceral adipose tissue, and abdominal subcutaneous adipose tissue in the tirzepatide groups [63].

Although these findings explore surrogate endpoints for liver disease, the results offer a promising future for tirzepatide in NASH and NAFLD populations. A randomized, double-blind, placebo-controlled phase 2 study comparing the efficacy and safety of tirzepatide versus placebo in 196 patients with nonalcoholic steatohepatitis (SYNERGY-NASH) with or without diabetes is currently underway and is estimated to complete in 2024. The primary outcome is the percentage of participants with absence of NASH with no worsening of fibrosis on liver histology [64].

With improved weight loss and the additional pleiotropic benefits of GIP, other metabolic risk factors are improved with tirzepatide. In a post-hoc analysis of the published SURPASS trials, the change in systolic blood pressure was greater for tirzepatide versus the comparators of the trials (tirzepatide 5 mg: -5.1 to -1.3 mmHg, tirzepatide 10 mg: -6.5 to -1.7 mmHg, tirzepatide 15 mg: -11.5 to -3.1 mmHg) [65]. The authors stated changes in SBP were primarily, but not solely, mediated by weight loss showing a weak (r = 0.18 – 0.22), but significant (p< 0.001), correlation between changes in body weight and SBP [65]. Additionally, a difference was seen between some doses of tirzepatide and semaglutide 1 mg when SURPASS-2 was reviewed. The 10 mg dose of tirzepatide showed greater weight reduction versus semaglutide 1 mg by -1.8 mmHg (-3.4 to -0.1) while the 15 mg dose of tirzepatide reduced an additional -3 mmHg (-4.6 to -1.3) compared to semaglutide [65].

Tirzepatide also showed improvements in lipids and cardiovascular risk factors compared to dulaglutide 1.5 mg and placebo. In a double-blind, placebo-controlled, phase 2b trial, patients with diabetes were administered all doses of tirzepatide, dulaglutide, or placebo. Blood samples of these patients were collected at baseline, week 4, week 12, and week 26. At week 26, LDL-C was reduced by 19.0% (-36.0% to -1.9%, p=.029) in the tirzepatide 15 mg group compared to placebo while it was reduced by 17.8% (-33.7% to -2.0%, p= 0.028) in the dulaglutide 1.5 mg group compared to placebo though differences between the 2 medications were not seen [60]. Triglycerides were significantly reduced at all doses of tirzepatide compared to placebo. Additionally, along with lowering triglycerides against placebo, tirzepatide 10 mg (-23.6%, CI: -34.3% to -10.5%, p<.001) and 15 mg (-27.9%, CI: -38.9% to -15.0%, p<.001) reduced triglycerides compared to dulaglutide 1.5 mg [60]. HDL-C did not change between tirzepatide and either placebo or dulaglutide [60]. Tirzepatide 10 and 15 mg also decreased large triglyceride-rich lipoprotein particles, small low-density lipoprotein particles, and lipoprotein insulin resistance score [60]. Finally, the study found that the largest predictor of triglyceride lowering in the tirzepatide groups was change in apoC-III levels and not body weight [60].

Lastly, a post-hoc analysis of the aforementioned 26-week trial analyzed biomarkers to assessed insulin sensitivity and beta-cell function. Homeostatic model assessment (HOMA) 2-B provides a validated tool to assess beta-cell function. The study showed significant decreases in HOMA2-B scores, proinsulin/insulin ratios, and proinsulin/C-peptide ratios in both tirzepatide (all doses) and dulaglutide compared to placebo [66]. Additionally, tirzepatide 10 mg decreased HOMA2-IR, a tool that measures insulin resistance, compared to both placebo and dulaglutide [66]. Multiple linear regression analysis showed that weight loss significantly (p<.028) explained only 13% of HOMA2-IR improvement for tirzepatide 10 mg and only 21% of improvement for tirzepatide 15 mg [66].

### Tirzepatide effects: adverse effects of tirzepatide

Gastrointestinal adverse effects of tirzepatide are similar to GLP-1 RAs, which include nausea and vomiting likely caused by the appetite-suppressing mechanism of incretins [47]. Other common adverse effects include diarrhea, constipation, dyspepsia, abdominal pain, and decreased appetite [47]. 6.6% of patients discontinue tirzepatide due to adverse effects during its phase 3 trial [47]. It is important to note that as with GLP-1 RAs, adverse effects of tirzepatide are dose-dependent. Hypoglycemia is a potential adverse effect though this is rare given the glucose-dependent mechanism and is usually seen in patients taking insulin or insulin secretagogues [47]. Other rare adverse effects include pancreatitis and cholelithiasis; the medication should be used with caution in patients with a history of either of these conditions [45]. Adverse effects are similar in patients using the medication for diabetes to those using the medication for weight loss [67]. Finally, similar to GLP-1 RAs, the medication should not be used in patients with a history of medullary thyroid carcinoma (MTC) or Multiple Endocrine Neoplasia syndrome type 2 (MEN 2) [31, 43].

### Cost-comparison of tirzepatide

Although very effective, GLP-1 RA’s are among the highest cost class of medications for the treatment of diabetes. Tirzepatide has a slightly higher cost than GLP-1 RAs for diabetes. Although cost to patient will differ based on insurance, Table 4 shows the average wholesale price (AWP) of tirzepatide compared to other diabetes drug classes. One example from each of the common diabetes classes is listen in Table 4 since other medications in the same class typically have similar costs. Despite likely being a similar tier as GLP-1 RAs and SGLT2is for many patient insurances, this elevated AWP is important to note for patients with co-insurances, deductibles, the Medicare coverage gap, patients without insurance, and for the total cost of healthcare.

**Table 4:** Lowest Average Wholesale Price (AWP) of a Sample of Antidiabetic Agents

**Medication**	**Average Wholesale Price (28 day supply)**
Tirzepatide	$1,169.20
Semaglutide SC (GLP-1 RA)	$1,070.46
Semaglutide PO (GLP-1 RA)	$999.04
Empagliflozin (SGLT-2i)	$638.96
Metformin^a^ (biguanide)	$1.96
Glipizide^a^ (sulfonylurea)	$16.80
Pioglitazone^a^ (thiazolidinedione)	$2.80

^a^Prices of generics are variable. The lowest generic AWP is shown

[68]

Table 5 also shows the cost of tirzepatide compared to other weight loss medications. It is important to recall that tirzepatide for weight loss is not yet approved and this table assumes that the cost of tirzepatide for weight loss will be the same as the cost for diabetes though this may not be as in the case of semaglutide. It is also important to note that combination weight loss products are brand name but are sometimes split into their generics by practitioners, an off-label method of getting similar doses and weight loss at a lower cost.

**Table 5:** Lowest Average Wholesale Price (AWP) of FDA-Approved Weight Loss Agents

**Medication**	**Price (28 day supply)**
Tirzepatide^b^	$1,169.20
Semaglutide	$1,618.84
Phentermine/topiramate	$223.44
Bupropion/naltrexone	$175
Orlistat (Rx)	$242.76
Orlistat (OTC)	$16.52

^b^This table assumes that the cost of tirzepatide for weight will be the same as the cost for diabetes

[68]

## Concluding remarks, place in therapy, and future perspective

Tirzepatide is a novel agent that is approved for the treatment of type 2 diabetes and will likely be approved for weight loss. Its novel mechanism allows the peptide to act as an agonist for both GLP-1 and GIP receptors. The clinical effects of the medication are similar to GLP-1 RAs. Although comparison studies with GLP-1 RA's have been conducted in the past, new doses have since been approved, making it difficult to compare tirzepatide to GLP-1 RA's based on previous studies. However, tirzepatide seems to be at least as potent as GLP-1 RAs at lowering blood sugar in those with diabetes. The larger benefit with the additional agonism of GIPr seems to be its effect on weight loss. Tirzepatide has been shown to be as potent or more potent than GLP-1 RAs in patients with or without diabetes. Although not yet completed, cardiovascular outcomes of tirzepatide will likely be similar to GLP-1 RAs based on the mechanism of the drug. Adverse effects of tirzepatide are similar to GLP-1 RAs.

This novel medication will likely be used in instances similar to GLP-1 RAs especially in patients with diabetes with high A1Cs and patients with diabetes with a compelling need to lose weight. Until further evidence is provided, GLP-1 RAs will likely be used preferentially in patients with cardiovascular disease or cardiovascular risk factors. Once studied, the cardiovascular effects of tirzepatide will likely be similar to GLP-1 RAs. Patients without diabetes but with obesity will likely be eligible for using this medication especially if the patient is looking for a potent agent with no stimulatory adverse effects. While this medication remains branded, its use will be limited by its cost. Future research will focus on the benefit of tirzepatide in patients with chronic kidney disease and non-alcoholic fatty liver disease. If cardiovascular outcomes of tirzepatide are similar to GLP-1 RAs, the medication may be used by practitioners preferentially prior to using GLP-1 RAs in patients with or without diabetes who desire weight-loss. Further research will clarify tirzepatide’s place in therapy.

## Data Availability

All data on tirzepatide are included within this paper and its supplementary information files.

## References

[1] Kim W, Egan JM (2008). The role of incretins in glucose homeostasis and diabetes treatment. Pharmacol Rev.

[2] Yabe D, Seino Y (2011). Two incretin hormones GLP-1 and GIP: Comparison of their actions in insulin secretion and β cell preservation. Prog Biophys Mol Biol.

[3] Yip RGC, Wolfe MM (1999). Gif biology and fat metabolism. Life Sci.

[4] Ross SA, Dupre J (1978). Effects of ingestion of triglyceride or galactose on secretion of gastric inhibitory polypeptide and on responses to intravenous glucose in normal and diabetic subjects. Diabetes.

[5] Trümper A, Trümper K, Trusheim H, Arnold R, Göke B, Hörsch D (2001). Glucose-dependent insulinotropic polypeptide is a growth factor for β (INS-1) cells by pleiotropic signaling. Mol Endocrinol.

[6] Gupta K, Raja A. Physiology, Gastric Inhibitory Peptide. 2022 Sep 26. In: StatPearls [Internet]. Treasure Island (FL): StatPearls Publishing; 2023 Jan–. PMID:31536259. 31536259

[7] Willms B, Werner J, Holst JJ, Orskov C, Creutzfeldt W, Nauck MA (1996). Gastric emptying, glucose responses, and insulin secretion after a liquid test meal: Effects of exogenous glucagon-like peptide-1 (GLP-1)-(7–36) amide in type 2 (noninsulin-dependent) diabetic patients. J Clin Endocrinol Metab.

[8] Komatsu R, Matsuyama T, Namba M, Watanabe N, Itoh H, Kono N, Tarui S (1989). Glucagonostatic and insulinotropic action of glucagonlike peptide i-(7–36)-amide. Diabetes.

[9] Prigeon RL, Quddusi S, Paty B, D'Alessio DA (2003) Suppression of glucose production by GLP-1 independent of islet hormones: A novel extrapancreatic effect. Am J Physiol-Endocrinol Metab. 285(4). 10.1152/ajpendo.00024.2003 10.1152/ajpendo.00024.2003 12773303

[10] Farilla L, Hui H, Bertolotto C, Kang E, Bulotta A, Di Mario U, Perfetti R (2002). Glucagon-like peptide-1 promotes islet cell growth and inhibits apoptosis in Zucker diabetic rats. Endocrinology.

[11] Perfetti R, Zhou J, Doyle ME, Egan JM (2000). Glucagon-like peptide-1 induces cell proliferation and pancreatic-duodenum homeobox-1 expression and increases endocrine cell mass in the pancreas of old, glucose-intolerant rats. Endocrinology.

[12] Holst JJ (2013). Incretin hormones and the satiation signal. Int J Obes.

[13] Samms RJ, Coghlan MP, Sloop KW (2020). How may GIP enhance the therapeutic efficacy of GLP-1?. Trends Endocrinol Metab.

[14] Drucker DJ (2001). Glucagon-like peptide 21. J Clin Endocrinol Metab.

[15] Aroda VR, Blonde L, Pratley RE (2022). A new era for oral peptides: Snac and the development of oral semaglutide for the treatment of type 2 diabetes. Rev Endocr Metab Disord.

[16] Nauck MA, Vilsbøll T, Gallwitz B, Garber A, Madsbad S (2009) Incretin-based therapies. Diabetes Care. 32(suppl_2). 10.2337/dc09-s315 10.2337/dc09-S315 PMC2811437 19875556

[17] Brown JC, Dryburgh JR (1971). A gastric inhibitory polypeptide II: The complete amino acid sequence. Can J Biochem.

[18] Vollmer K, Holst JJ, Baller B, Ellrichmann M, Nauck MA, Schmidt WE, Meier JJ (2008). Predictors of incretin concentrations in subjects with normal, impaired, and diabetic glucose tolerance. Diabetes.

[19] Nauck MA, Vardarli I, Deacon CF, Holst JJ, Meier JJ (2010). Secretion of glucagon-like peptide-1 (GLP-1) in type 2 diabetes: What is up, what is down?. Diabetologia.

[20] Ross SA, Brown JC, Dupré J (1977). Hypersecretion of gastric inhibitory polypeptide following oral glucose in diabetes mellitus. Diabetes.

[21] Zhou J, Livak MF, Bernier M, Muller DC, Carlson OD, Elahi D, Maudsley S, Egan JM (2007) Ubiquitination is involved in glucose-mediated downregulation of GIP receptors in islets. Am J Physiol-Endocrinol Metab. 293(2). 10.1152/ajpendo.00070.2007 10.1152/ajpendo.00070.2007 PMC2640485 17505054

[22] Holz GG, Kiihtreiber WM, Habener JF (1993). Pancreatic beta-cells are rendered glucose-competent by the insulinotropic hormone glucagon-like peptide-1(7–37). Nature.

[23] Hui H, Farilla L, Merkel P, Perfetti R (2002) The short half-life of glucagon-like peptide-1 in plasma does not reflect its long-lasting beneficial effects. Eur J Endocrinol. 863–869. 10.1530/eje.0.1460863 10.1530/eje.0.1460863 12039708

[24] Fadini GP, Bottigliengo D, D’Angelo F, Cavalot F, Bossi AC, Zatti G, Baldi I, Avogaro A (2018). Comparative effectiveness of DPP-4 inhibitors versus sulfonylurea for the treatment of type 2 diabetes in routine clinical practice: A retrospective multicenter real-world study. Diabetes Ther.

[25] Deane AM, Chapman MJ, Horowitz M (2010). The therapeutic potential of a venomous lizard: The use of glucagon-like peptide-1 analogues in the critically ill. Crit Care.

[26] Bond A (2006). Exenatide (Byetta) as a novel treatment option for type 2 diabetes mellitus. Bayl Univ Med Cent Proc.

[27] Bydureon [package insert]. Wilmington, DE: AstraZeneca; 2018.

[28] Ozempic [package insert]. Bagsvaerd, Denmark: NovoNordisk; 2017.

[29] Rybelsus [package insert]. Bagsvaerd, Denmark: NovoNordisk; 2022.

[30] Trulicity [package insert]. Indianapolis, IN: Eli Lilly; 2022.

[31] Victoza [package insert]. Bagsvaerd, Denmark: NovoNordisk; 2017.

[32] Deng Y, Polley EC, Wallach JD, Dhruva SS, Herrin J, Quinto K, Gandotra C, Crown W, Noseworthy P, Yao X, Lyon TD, Shah ND, Ross JS, McCoy RG (2022). Emulating the grade trial using Real World Data: Retrospective Comparative Effectiveness Study. BMJ.

[33] Saxenda [package insert]. Bagsvaerd, Denmark: NovoNordisk; 2014.

[34] Wegovy [package insert]. Bagsvaerd, Denmark: NovoNordisk; 2022.

[35] Gerstein HC, Colhoun HM, Dagenais GR, et al. Dulaglutide and cardiovascular outcomes in type 2 diabetes (Rewind): a double-blind, randomised placebo-controlled trial. Lancet. 2019;394(10193):121–30. 10.1016/S0140-6736(19)31149-3 31189511

[36] Marso SP, Bain SC, Consoli A (2016). Semaglutide and cardiovascular outcomes in patients with type 2 diabetes. N Engl J Med..

[37] Marso SP, Daniels GH, Brown-Frandsen K (2016). Liraglutide and cardiovascular outcomes in type 2 diabetes. N Engl J Med..

[38] ElSayed NA, Aleppo G, Aroda VR, Bannuru RR, Brown FM, Bruemmer D, Collins BS, Cusi K, Das SR, Gibbons CH, Giurini JM, Hilliard ME, Isaacs D, Johnson EL, Kahan S, Khunti K, Kosiborod M, Leon J, Lyons SK, Gabbay RA (2022) Standards of care in diabetes—2023. Diabetes Care. 10.2337/dc23-sint

[39] Heerspink HJL, Stefánsson BV, Correa-Rotter R, Chertow GM, Greene T, Hou F-F, Mann JFE, McMurray JJV, Lindberg M, Rossing P, Sjöström CD, Toto RD, Langkilde A-M, Wheeler DC (2020). Dapagliflozin in patients with chronic kidney disease. N Engl J Med.

[40] The EMPA-KIDNEY Collaborative Group (2022). Empagliflozin in patients with chronic kidney disease. N Engl J Med.

[41] Watson, J. D. (n.d.). Carmot Therapeutics closes $160 million series D financing to Advance Clinical Pipeline of Novel Incretin receptor modulators. BioSpace. https://www.biospace.com/article/releases/carmot-therapeutics-closes-160-million-series-d-financing-to-advance-clinical-pipeline-of-novel-incretin-receptor-modulators/

[42] Ludvik B, Giorgino F, Jódar E, Frias JP, Fernández Landó L, Brown K, Bray R, Rodríguez Á. Once-weekly tirzepatide versus once-daily insulin degludec as add-on to metformin with or without SGLT2 inhibitors in patients with type 2 diabetes (surpass-3): A randomised, open-label, parallel-group, phase 3 trial. Lancet. 2021;398(10300):583–98. 10.1016/s0140-6736(21)01443-4. 10.1016/S0140-6736(21)01443-4 34370970

[43] Mounjaro [package insert]. Indianapolis, IN: Eli Lilly; 2022.

[44] Dahl D, Onishi Y, Norwood P (2022). Effect of Subcutaneous Tirzepatide vs Placebo Added to Titrated Insulin Glargine on Glycemic Control in Patients With Type 2 Diabetes: The SURPASS-5 Randomized Clinical Trial. JAMA..

[45] Del Prato S, Kahn SE, Pavo I, et al. SURPASS-4 Investigators. Tirzepatide versus insulin glargine in type 2 diabetes and increased cardiovascular risk (SURPASS-4): a randomised, open-label, parallel-group, multicentre, phase 3 trial. Lancet 2021;398:1811–1824. 10.1016/S0140-6736(21)02188-7 34672967

[46] Frías JP, Davies MJ, Rosenstock J (2021). Tirzepatide versus Semaglutide Once Weekly in Patients with Type 2 Diabetes. N Engl J Med..

[47] Rosenstock J, Wysham C, Frías JP, Kaneko S, Lee CJ, Fernández Landó L, Mao H, Cui X, Karanikas CA, Thieu VT. Efficacy and safety of a novel dual GIP and GLP-1 receptor agonist tirzepatide in patients with type 2 diabetes (surpass-1): A double-blind, randomised, phase 3 trial. Lancet. 2021;398(10295):143–55. 10.1016/s0140-6736(21)01324-6. 10.1016/S0140-6736(21)01324-6 34186022

[48] A randomized, phase 3, open-label trial comparing the effect of the addition of tirzepatide once weekly versus insulin lispro (U100) three times daily in participants with type 2 diabetes inadequately controlled on insulin glargine (U100) with or without metformin (SURPASS-6). October 19, 2021.

[49] Hernandez AF, Green JB, Janmohamed S, et al. Albiglutide and cardiovascular outcomes in patients with type 2 diabetes and cardiovascular disease (Harmony outcomes): a double-blind, randomised placebo-controlled trial. Lancet. 2018;392(10157):1519–29. 10.1016/S0140-6736(18)32261-X 30291013

[50] Frias JP, Nauck MA, Van J (2018). Efficacy and safety of LY3298176, a novel dual GIP and GLP-1 receptor agonist, in patients with type 2 diabetes: a randomised, placebo-controlled and active comparator-controlled phase 2 trial. Lancet..

[51] Garvey WT, Frias JP, Jastreboff AM, le Roux CW, Sattar N, Aizenberg D, Mao H, Zhang S, Ahmad NN, Bunck MC, Benabbad I, Zhang XM, Abalos FH, Manghi FCP, Zaidman CJ, Vico ML, Aizenberg D, Costanzo PR, Serra LP, Jones T (2023). Tirzepatide once weekly for the treatment of obesity in people with type 2 diabetes (surmount-2): A double-blind, randomised, multicentre, placebo-controlled, phase 3 trial. The Lancet.

[52] Tchang BG, Aras M, Kumar RB, et al. Pharmacologic Treatment of Overweight and Obesity in Adults. [Updated 2021 Aug 2]. In: Feingold KR, Anawalt B, Boyce A, et al., editors. Endotext [Internet]. South Dartmouth (MA): MDText.com, Inc.; 2000-. Available from: https://www.ncbi.nlm.nih.gov/books/NBK279038/25905267

[53] Zelniker TA, Wiviott SD, Raz I (2019). Comparison of the effects of glucagon-like peptide receptor agonists and sodium-glucose cotransporter 2 inhibitors for prevention of major adverse cardiovascular and renal outcomes in type 2 diabetes mellitus. Circulation.

[54] Zweck E, Roden M. GLP-1 receptor agonists and cardiovascular disease: drug-specific or class effects? Lancet Diabetes Endocrinol. 2019;7:89–90 [PubMed]. 10.1016/S2213-8587(18)30351-6 30683221

[55] Drucker DJ (2016). The cardiovascular biology of glucagon-like peptide-1. Cell Metab.

[56] Koska J, Sands M, Burciu C (2015). Exenatide protects against glucose- and lipid-induced endothelial dysfunction: evidence for direct vasodilation effect of GLP-1 receptor agonists in humans. Diabetes.

[57] Robinson LE, Holt TA, Rees K, Randeva HS, O’Hare JP (2013). Effects of exenatide and liraglutide on heart rate, blood pressure and body weight: systematic review and meta-analysis. BMJ Open.

[58] Wang B, Zhong J, Lin H (2013). Blood pressure-lowering effects of GLP-1 receptor agonists exenatide and liraglutide: a meta-analysis of clinical trials. Diabetes Obes Metab.

[59] Wilson JM, Lin Y, Luo MJ (2022). The dual glucose-dependent insulinotropic polypeptide and glucagon-like peptide-1 receptor agonist tirzepatide improves cardiovascular risk biomarkers in patients with type 2 diabetes: A post hoc analysis. Diabetes Obes Metab.

[60] Wilson JM, Nikooienejad A, Robins DA (2020). The dual glucose-dependent insulinotropic peptide and glucagon-like peptide-1 receptor agonist, tirzepatide, improves lipoprotein biomarkers associated with insulin resistance and cardiovascular risk in patients with type 2 diabetes. Diabetes Obes Metab..

[61] Sattar N, McGuire DK, Pavo I, et al. Tirzepatide cardiovascular event risk assessment: a pre-specified meta-analysis. Nat Med. 2022;28:591–598F. 10.1038/s41591-022-01707-4 PMC8938269 35210595

[62] Hartman ML, Sanyal AJ, Loomba R, Wilson JM, Nikooienejad A, Bray R, Karanikas CA, Duffin KL, Robins DA, Haupt A (2020). Effects of novel dual GIP and GLP-1 receptor agonist Tirzepatide on biomarkers of nonalcoholic steatohepatitis in patients with type 2 diabetes. Diabetes Care.

[63] Gastaldelli A, Cusi K, Fernández Landó L, Bray R, Brouwers B, Rodríguez Á (2022). Effect of tirzepatide versus insulin degludec on liver fat content and abdominal adipose tissue in people with type 2 diabetes (surpass-3 MRI): A substudy of the randomised, open-label, parallel-group, phase 3 surpass-3 trial. Lancet Diabetes Endocrinol.

[64] A Study of Tirzepatide (LY3298176) in Participants With Nonalcoholic Steatohepatitis (NASH) - Full Text View - ClinicalTrials.gov (n.d.). https://clinicaltrials.gov/ct2/show/NCT04166773

[65] Lingvay I, Mosenzon O, Brown K, Cui X, O’Neill C, Fernández Landó L, Patel H. Systolic blood pressure reduction with tirzepatide in patients with type 2 diabetes: Insights from surpass clinical program. Cardiovasc Diabetol 2023;22(1). 10.1186/s12933-023-01797-5 10.1186/s12933-023-01797-5 PMC10039543 36964557

[66] Thomas MK, Nikooienejad A, Bray R, Cui X, Wilson J, Duffin K, Milicevic Z, Haupt A, Robins DA (2020). Dual GIP and GLP-1 receptor agonist Tirzepatide improves beta-cell function and insulin sensitivity in type 2 diabetes. J Clin Endocrinol Metab.

[67] Jastreboff AM, Aronne LJ, Ahmad NN, Wharton S, Connery L, Alves B, Kiyosue A, Zhang S, Liu B, Bunck MC, Stefanski A (2022). Tirzepatide once weekly for the treatment of obesity. N Engl J Med.

[68] Uptodate: Industry-leading clinical decision support. Back to top (n.d.). Retrieved January 5, 2023, from https://www.uptodate.com/

